# Structured reporting of chest CT in COVID-19 pneumonia: a consensus proposal

**DOI:** 10.1186/s13244-020-00901-7

**Published:** 2020-08-12

**Authors:** E. Neri, F. Coppola, A. R. Larici, N. Sverzellati, M. A. Mazzei, P. Sacco, G. Dalpiaz, B. Feragalli, V. Miele, R. Grassi

**Affiliations:** 1 Diagnostic and Interventional Radiology, Department of Translational Research, Università degli Studi di Pisa, Radiodiagnostica 3, Via Roma 67 –, 56126 Pisa, SD Italy; 2 Malpighi Radiology Unit, Department of Diagnostic and Preventive Medicine, University Hospital of Bologna Sant’Orsola-Malpighi Polyclinic, Bologna, Italy; 3 Section of Radiology, Department of Radiological and Hematological Sciences, Catholic University of the Sacred Heart Rome Campus, “Agostino Gemelli” University Polyclinic Foundation IRCCS, Roma, Italy; 4 Division of Radiology, Department of Medicine and Surgery, University of Parma, Parma, Italy; 5 Department of Medical, Surgical and Neuro Sciences, Diagnostic Imaging, University of Siena, Azienda Ospedaliera Universitaria Senese, Siena, Italy; 6 Diagnostic Imaging Unit, Department of Medical, Surgical and Neuro Sciences, Azienda Ospedaliera Universitaria Senese, Siena, Italy; 7 Department of Radiology, Bellaria Carlo Alberto Pizzardi Hospital, Bologna, Italy; 8 Department of Medical, Oral and Biotechnological Sciences, University G. d’Annunzio Chieti-Pescara, Chieti, Italy; 9 Department of Radiology, Azienda Ospedaliero Universitaria Careggi, Firenze, Italy; 10 Department of Clinical and Experimental Medicine, “F. Magrassi-A. Lanzara”, University of Campania Luigi Vanvitelli, Naples, Italy

**Keywords:** COVID-19, Structured reporting, Computed tomography

## Abstract

**Objectives:**

The need of a standardized reporting scheme and language, in imaging of COVID-19 pneumonia, has been welcomed by major scientific societies. The aim of the study was to build the reporting scheme of chest CT in COVID-19 pneumonia.

**Methods:**

A team of experts, of the Italian Society of Medical and Interventional Radiology (SIRM), has been recruited to compose a consensus panel. They used a modified Delphi process to build a reporting scheme and expressed a level of agreement for each section of the report. To measure the internal consistency of the panelist ratings for each section of the report, a quality analysis based on the average inter-item correlation was performed with Cronbach’s alpha (Cα) correlation coefficient.

**Results:**

The overall mean score of the experts and the sum of score were 3.1 (std.dev. ± 0.11) and 122 in the second round, and improved to 3.75 (std.dev. ± 0.40) and 154 in the third round. The Cronbach’s alpha (Cα) correlation coefficient was 0.741 (acceptable) in the second round and improved to 0.789 in the third round. The final report was built in the management of radiology report template (MRRT) and includes *n* = 4 items in the procedure information, *n* = 5 items in the clinical information, *n* = 16 in the findings, and *n* = 3 in the impression, with overall 28 items.

**Conclusions:**

The proposed structured report could be of help both for expert radiologists and for the less experienced who are faced with the management of these patients. The structured report is conceived as a guideline, to recommend the key items/findings of chest CT in COVID-19 pneumonia.

## Key points

- The structured report includes all potential findings at CT in COVID-19 pneumonia.
- The structured report is compliant with the recommendations of major scientific societies for reporting and the use of a standard language in chest CT of COVID-19 pneumonia.
- The structured report is compliant with the management of radiology report template standard and shareable in html format.

## Introduction

The novel coronavirus (SARS-cov-2) infection outbreak, rapidly spreading from Wuhan City (Hubei Province, China) to extra continental countries since December 2019, has been declared a pandemic by the World Health Organization (WHO) on March 11, 2020. In February 2020, the epidemic exploded in Italy, with an exponential increase in the number of cases, following a curve quite similar to that observed in China [1].

At the time of this writing, Italy is the eleventh country in the world by number of confirmed cases and the fourth by number of deaths [2, 3].

The COVID-19 pandemic has forced the radiology department to re-organize their logistic and workload, giving priority to the management of these patients. Basically, dedicated COVID-paths have been set on to avoid contact between infected and non-infected patients [4]. Several major radiological scientific societies have published guidelines on the diagnostic work-up of suspected or ascertained COVID-19 patients, suggesting the use of imaging on the basis of the clinical findings [5–7].

However, there is still no consensus about the use of chest x-ray (CXR) or computed tomography (CT) as first-line imaging tools. The British Society of Thoracic Imaging (BSTI) considers chest radiography as a key decision tool for suspected COVID-19 pneumonia, as well as the Italian Society of Medical and Interventional Radiology (SIRM) that suggest CXR as the first modality of choice and CT as second level modality. The American College of Radiology (ACR) discourages the routinary use of CT since the high risk of spreading the infection among patients and healthcare personnel. The European Society of Radiology (ESR) and the European Society of Thoracic Imaging (ESTI) suggest that CXR should not be used as the first-line technique and should be restricted to the follow-up of patients admitted to intensive care units, who are too fragile to be sent to CT; however, both suggest that CT is indicated only when the degree of severity of respiratory symptoms justify the investigation [8]. Recently, also the World Health Organization delivered guidelines on chest imaging in COVID-19 and found on a meta-analysis that when used for diagnosis in symptomatic patients, negative CT results are more useful for diagnosis than positive results [9]. Therefore, a definite diagnostic flow chart cannot be outlined at this stage, and further data are required to hypothesize sufficient or optimal technical imaging requirements for the hospitals.

Nevertheless, beyond the imaging workflow, of not less importance is the way the imaging results are reported. As COVID-19 imaging patterns are non-specific, it is difficult to reach consistent conclusions in the free text radiological report. Variability among reports may increase the uncertainty on the diagnosis, but also on the estimation of disease severity, which is of great importance for the therapeutic management of these patients.

The need of a uniform and standardized reporting scheme and language, in imaging of COVID-19 pneumonia, has been welcomed by major scientific societies [10–13]. In the midst of the pandemic, SIRM has promoted the COVID-19 structured reporting initiative for chest CT, to stimulate a uniform reporting strategy and harmonize the radiological reports of imaging departments across the country [14]. The aim of the study was to build the reporting scheme of chest CT in COVID-19 pneumonia, on the basis of a consensus among experts in thoracic imaging and imaging informatics.

## Materials and methods

In 2018, in adherence with the RSNA (Radiological Society of North America) and ESR structured reporting initiative, SIRM launched the Italian initiative aimed at creating a repository of structured reports, available to its members, to be used in clinical practice [15, 16]. The initiative has been primarily focused on oncologic imaging, and a panel of experts, recruited from the SIRM study sections or chapters, has been set up. All panelists worked in a collaborative fashion in clusters of expertise, i.e., cancer of the GI tract, MSK, and abdomen.

In view of the COVID-19 pandemic, a team of experts, composed by members of the college of thoracic radiology and imaging informatics of SIRM, and society leadership, has been recruited to build a focused working group (A.L., N.S., F.C., B.F., G.D., P.S., M.A.M., E. N.) on drafting a chest CT COVID-19 pneumonia structured report. One additional panelist, who did not express a vote, was chosen to play the role of facilitator (R.G.). The working group used a modified Delphi process to rate the level of agreement on each section of the reporting scheme.

Three Delphi rounds were conducted [17]. In a first round, each panel member participated independently in the drafting of the reporting scheme by email exchange and through online meetings. The panelists performed a review of the existing literature (at the time of the first round, then updated in the second and third rounds) on PubMed, Google Scholar, and Scopus databases. The reporting scheme was assembled on a Google document and shared among panelists.

In a second round, to evaluate the level of agreement of the panelists on the final draft of the structured report, a Google form questionnaire was delivered through email. Each panelist provided the level of agreement on specific sections of the report (procedure information, clinical information, findings, and impression) through a 4 point Likert scale (1-disagree entirely, 2-somewhat disagree, 3-somewhat agree, 4-agree entirely).

**Fig. 1:**
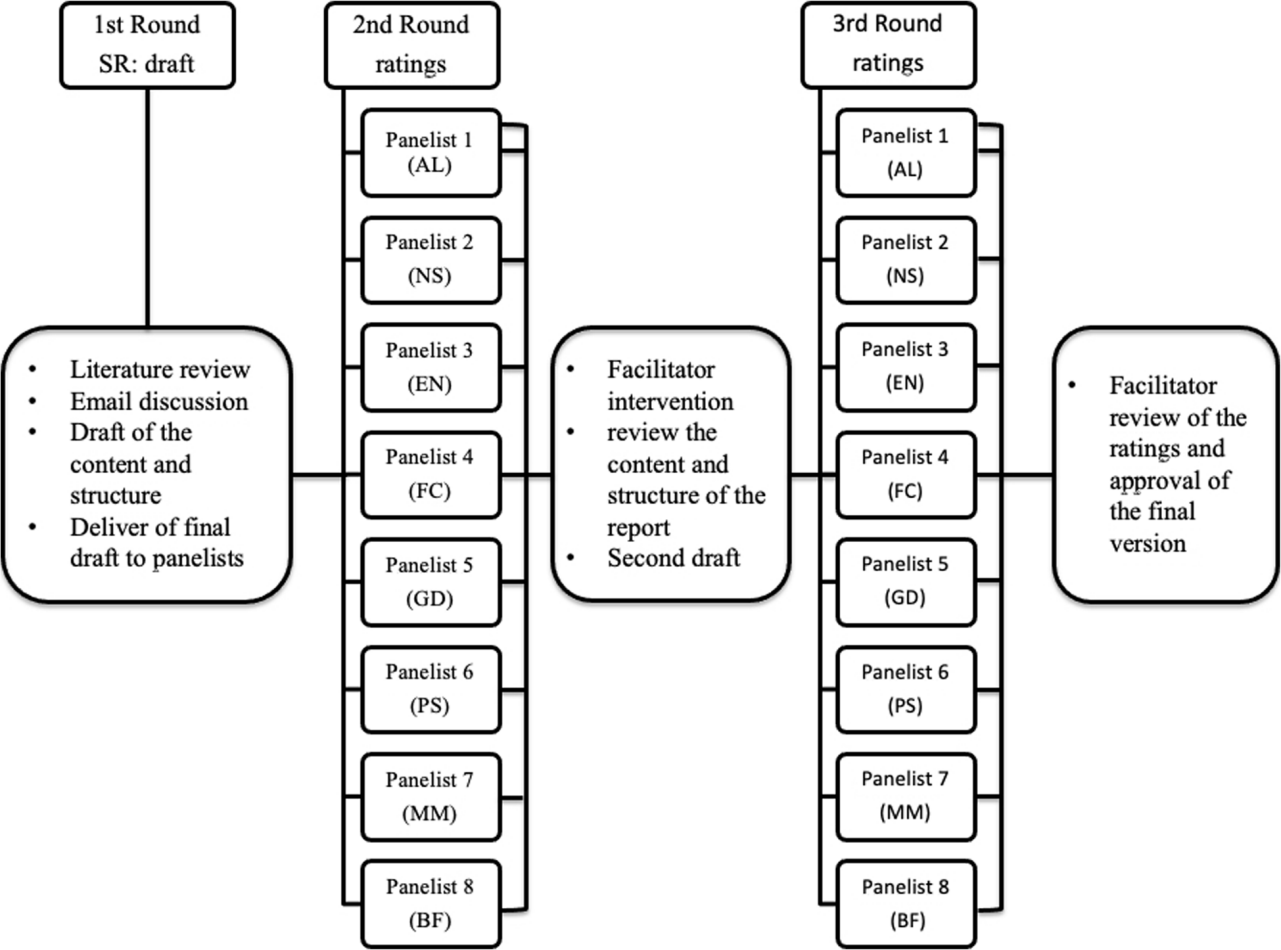
Flow chart of the Delphi consensus

After the second round, the facilitator collected the ratings from the panelists and calculated the mean score of agreement for each section. If the mean score was less than 3 or the panelists suggested further changes to the format and content of the structured report, the facilitator proposed a reviewed version of the report to the panelists and started a second poll to reach a higher level of agreement (Fig. 1).

The final structured report, resulting from the third round, was assembled on the radreport.org website of RSNA, through the T-Rex template editor, freely available as open source software, in HTML format according to the IHE (Integrating Healthcare Enterprise) MRRT (management of radiology report templates) profile, which defines both the format of radiology reporting templates using an extension of Hypertext Markup Language version 5 (HTML5) and the transportation mechanism to query, retrieve, and store these templates [18]. The report was built through a sequence of “coded questions,” included in the predefined sections of the T-Rex editor [18].

### Statistical analysis

Answers from each panelist were exported in Microsoft Excel® format for ease of data collection and statistical analysis.

All ratings of panelists for each section were analyzed with descriptive statistics measuring the mean score, the standard deviation, and the sum of scores. A mean score of 3 was considered good and a score of 4 excellent.

To measure the internal consistency of the panelist ratings for each section of the report, a quality analysis based on the average inter-item correlation was performed with Cronbach’s alpha (Cα) correlation coefficient [19, 20]. The Cα test provides a measure of the internal consistency of a test or scale; it is expressed as a number between 0 and 1. Internal consistency describes the extent to which all the items in a test measure the same concept. Cα was determined after each round.

The closer Cα coefficient is to 1.0, the greater the internal consistency of the items in the scale. An alpha coefficient (α) > 0.9 was considered excellent, α > 0.8 good, α > 0.7 acceptable, α > 0.6 questionable, α > 0.5 poor, and α < 0.5 unacceptable. However, in the iterations an α of 0.8 was considered a reasonable goal for internal reliability.

All data were analyzed using the statistical package for social science (SPSS, Chicago, IL, USA).

## Results

### Consensus agreement

**Fig. 2:**
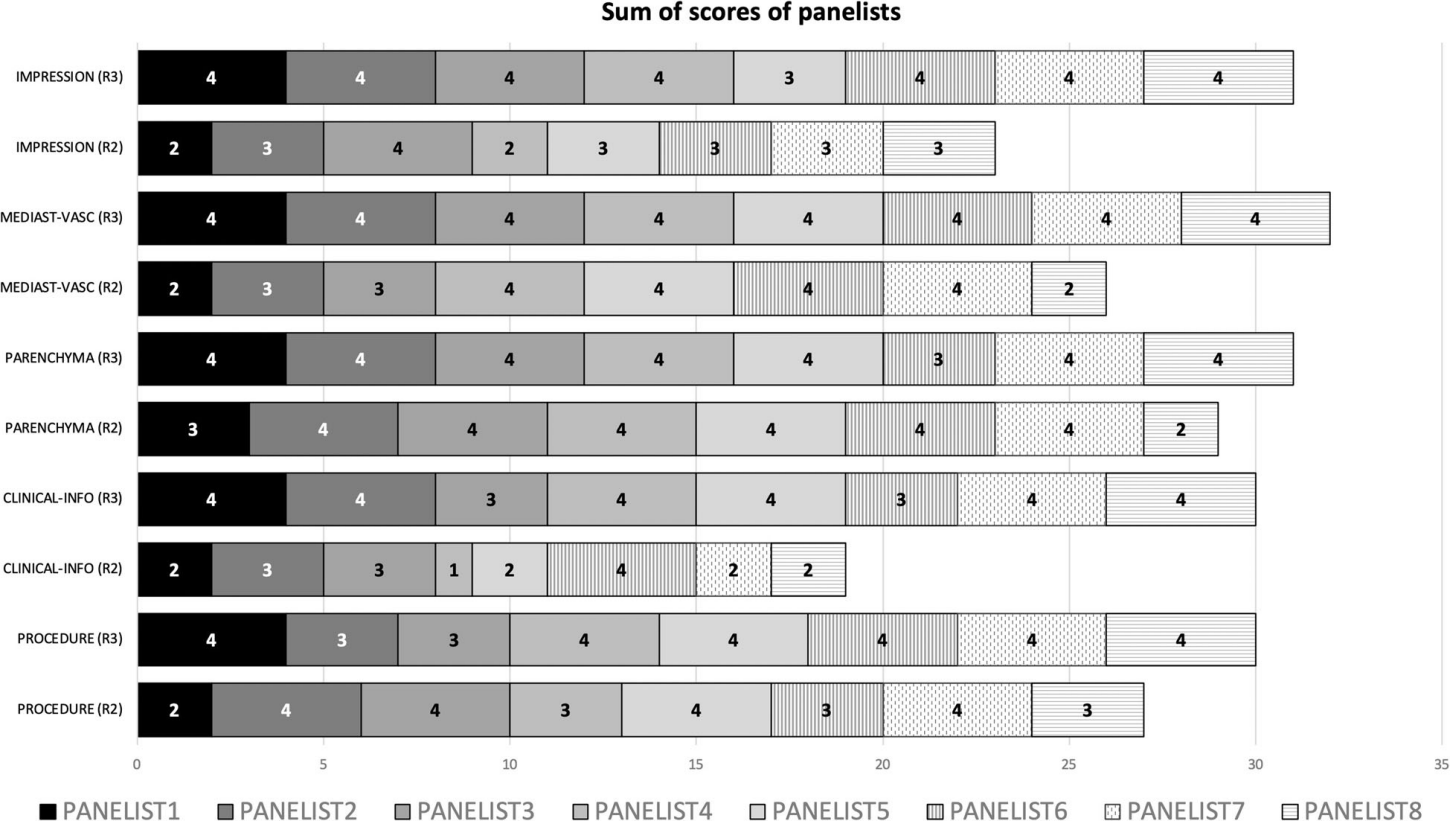
Sum of scores of each section of the structured report in round 2 (R2) and round 3 (R3)

In the second round, as reported in Table 1, all sections received more than a good rating, except the clinical information section and the impression section, which obtained a score of 2.3 and 2.8. However, in the third round, both improved to 3.1 and 4, respectively. The overall mean score of the experts and the sum of score were 3.1 (std.dev. ± 0.11) and 122 in the second round, and improved to 3.75 (std.dev. ± 0.40) and 154 in the third round (Table 1 and Fig. 2). The Cronbach’s alpha (Cα) correlation coefficient was 0.741 (acceptable) in the second round, and improved to 0.789 in the third round.

**Table 1:** Mean scores and sum of scores of panelists

	Mean scores and std.dev. and sum of scores by each section of the structured report Mean (std.dev.) Sum	
Structured report sections	Round 2	Round 3
Procedure information	3.37 (± 0.74) 27	3.50 (± 0.75) 30
Clinical information	2.37 (± 0.91) 19	3.1 (± 0.83) 30
Findings: parenchyma (GGO, consolidations, nodules, other)	3.62 (± 0.74) 29	3.75 (± 0.46) 31
Findings: mediastinum, vascular	3.25 (± 0.88) 26	4 32
Impression	2.87 (± 0.64) 21	3.87 (± 0.35) 31
Full structured report	3.1 (± 0.11) 122	3.7 (± 0.46) 154

### Structured report: format and content

**Fig. 3:**
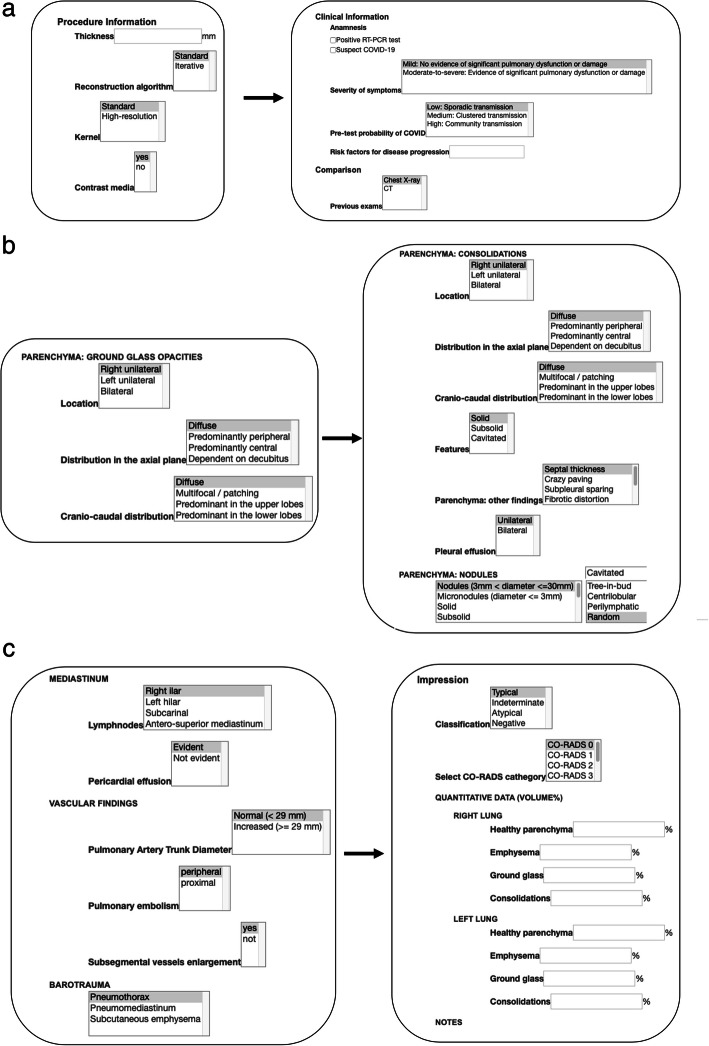
Structured report displayed in the MMRT format (www.radreport.org). **a** Sections “Procedure Information” and “Clinical Information”. **b** Section “Parenchyma”. **c** Sections “Mediastinum and Vascular findings”, and “Impression.” The subsection classification is based on the paper by Simpson et al. [13], as follows: Typical—peripheral, bilateral, GGO with or without consolidation or visible intralobular lines (crazy-paving). Multifocal GGO of rounded morphology with or without consolidation or visible intralobular lines (crazy-paving). Reverse halo sign or other findings of organizing pneumonia (seen later in the disease). Atypical—absence of typical or indeterminate features and presence of: isolated lobar or segmental consolidation without GGO. Discrete small nodules (centrilobular, tree in-bud). Lung cavitation. Smooth interlobular septal thickening with pleural effusion. Indeterminate—absence of typical features and presence of: multifocal, diffuse, perihilar, or unilateral GGO with or without consolidation lacking a specific distribution and are non-rounded or non-peripheral. Few very small GGO with a non-rounded and non-peripheral distribution. Negative—no CT features to suggest pneumonia. The subsection “Select CO-RADS category” is based on the paper by Prokop et al. [10], as follows: CO-RADS 0 not interpretable: scan technically insufficient for assigning a score; CO-RADS 1 very low: normal or non-infections; CO-RADS 2 low: typical for other infection but not COVID-19. CO-RADS 3 equivocal/unsure: features compatible with COVID-19, but also other diseases. CO-RADS 4 high: suspicious for COVID-19. CO-RADS 5 very high: typical for COVID-19. CO-RADS 6 proven: RT-PCR positive for SARS-CoV-2

The final report was built in the MRRT format (Fig. 3) and includes *n* = 4 items in the procedure information, *n* = 5 items in the clinical information, *n* = 16 in the findings, and *n* = 3 in the impression, with overall 28 items to fill. However, among them, the procedure and the clinical information have been set as recommended, as the radiologists will not be forced to fill the items during the reporting. The items of the impression section have been also left recommended, but the classification item was judged strongly recommended in order to provide suggestions on patient management. The CO-RADS category was included as optional for classification and statistical purposes. A quantitative data section, to include percentages of healthy parenchyma, emphysema, ground glass opacities, and consolidations, was included, in view of the emerging quantitative analysis tools available on the market, and to facilitate research and clinical trials data collection; however, even this section remains optional in the reporting scheme.

The structured report has been submitted to the radreport.org website of RSNA and is under review by the Template Library Advisory Panel (TLAP), a joint committee of RSNA and ESR. If approved and revised accordingly, it will be published.

## Discussion

The benefits of the structured report are well-known in the literature and already demonstrated by some clinical implementation trials [21–24].

Studies show that the structured report allows to standardize the communication of the findings, through a well-defined description scheme, a standard language with appropriate and shared terminology, and the possibility of providing quantitative data that can be used for data mining [25–27].

Despite the perception of these advantages by the radiological community, there are still many obstacles to clinical implementation, mainly related to the poor attitude to a change in the reporting methodology [28]. Moreover, despite the availability of an IHE MRRT profile that defines the format and the exchange protocol of templates, its integration in radiological information systems is quite rare as the literature reports few studies. Pinto dos Santos et al. converted in templates the free text reports of 521 consecutive cases which had been referred to the radiology department for CT pulmonary angiography with suspected pulmonary embolism [29]. Gichoya et al. describe the implementation of an open-source radiological information system that supports importing and use of IHE MRRT [30].

In the emergency situation of the pandemic from (SARS-cov-2) infection, CT plays an increasingly important role in the diagnostic workup of these patients, especially in those symptomatic with progressive worsening of symptoms [5]. In this context, it is important that the report includes mineable data, structured and meaningful to define the severity of the disease and address the therapeutic decision [11].

This was the motivation to develop a structured report for chest CT in suspected or confirmed COVID-19 patients, based on a consensus process by experts in the fields of thoracic radiology with the support of imaging informatics experts of SIRM.

The most debated sections during the composition of the structured report were those related to clinical information and impression, as clearly demonstrated by the low scores in the second round reported in Table 1. Indeed, in the absence of definite and shared guidelines, the clinical information might contain an extensive number of data (symptoms, laboratory data, respiratory function data, etc.) which are difficult, if not impossible, to be timely collected during interpretation and reporting of the clinical cases, both for the time necessary for collection and for the difficulty in a timely retrieval of such data from the hospital information system.

However, in the third round, there was a greater agreement among the panelists, probably explained by the availability of recent statements by international societies, as the multinational consensus statement of the Fleischner society (to which one of the panelists contributed). This statement bases the patient management on key components of common clinical scenarios, as the severity of symptoms, the pre-test probability, the age and comorbidities (as risk factors for disease progression), the disease progression, and the resource constraints [5]. Among these, severity of symptoms, pre-test probability, and risk factors were chosen because these are considered of major importance by the panel, thus limiting the length of the section of clinical information for the purposes of an easier use of the format in the clinical practice.

The section impression was also debated by the panelists as it was difficult to come to conclusions and suggestions at the time of the second round of the consensus. However, even in this case, more recent proposals of CT grading and categorical classifications of COVID-19 lung involvement have been published, as the Reporting and Data Systems with the COVID-RADS and the CO-RADS [10, 11], and the RSNA expert consensus statement on reporting chest CT findings related to COVID-19, endorsed also by the Society of Thoracic Radiology and the ACR [13].

The section findings are the key component of the report and derive both from a literature review of CT findings (primarily described by the Chinese researchers, as the first reports in the literature) and the experience acquired by the panelists, Italy being the second country affected by the pandemic with a large cohort of positive cases with lung involvement [31–35].

The description of parenchyma findings starts with the ground glass opacities, in terms of location, distribution in the axial plane (as the most immediate evaluation feasible at CT), and in the cranio-caudal plane (which could be evaluated by multiplanar reconstruction). Ground glass opacities are the main features of COVID-19 pneumonia [8, 34, 36, 37]. However, it is well-known that these findings are not typical of COVID-19 since these can be observed in other interstitial pneumonias [38–40].The same items used in the description of ground glass opacities were included in the consolidation subsection, with the addition of the features solid, subsolid, or cavitated. Other findings, non-specific of COVID-19 pneumonia, have been included, as septal thickening, crazy paving, subpleural sparing, fibrotic distortion, reversed halo sign, emphysema, and perilobular sign [8, 34, 41–43]. Although these findings (as well as ground glass opacities and consolidations) are not specific of COVID-19 infection, but can be observed in many interstitial lung diseases, we believe that the use of the structured report will allow for a detailed quantification of their incidence in these patients. In this context, the structured report, combined with artificial intelligence software, could help in the construction of predictive and prognostic models of disease, increasing the specificity of CT [44, 45]. Additional items regarding pulmonary nodules, if evident, were considered, using the typical classification into nodules and micronodules, with the solid, subsolid, cavitated, or tree-in-bud appearance, and the centrilobular, perilymphatic or random pattern of distribution, which are all non-specific of COVID-19 [35].

A subsection of the report is dedicated to the description of the vascular findings, as the pulmonary artery trunk diameter, the evidence of pulmonary embolism, and the subsegmental vessel enlargement. The attention to vascular findings has been raised up by the recent literature reports of thromboembolic complications. In a study by Lodigiani et al. on 388 consecutive patients with laboratory-proven COVID-19 infection, the incidence of thromboembolic complication was 7.7%, including pulmonary embolism, peripheral vein thrombosis, and stroke [46]. In patients admitted to intensive care units, the cumulative incidence of thromboembolic events rise up to 27% according to a study of Klok et al., of which pulmonary embolism represent the 80% of cases [47]. Such data suggest that in the case of suspicion of pulmonary embolism it would be recommended to perform a CT angiography study. Leonard-Lorant et al. found that 30% of the patients with COVID-19 infection were positive for acute pulmonary emboli on pulmonary CT angiograms [48].

The subsegmental vessel enlargement represents also a significant finding in COVID-19. In a study on chest CT features of COVID-19 pneumonia by Caruso et al., the reported rate of this finding on CT was 89% [35]. Moreover, Bai et al. report an incidence of subsegmental vascular enlargement of 58% in patients with COVID-19 pneumonia, versus 22% in those with other viral non-COVID-19 pneumonia, to support the hypothesis that this finding could be a more specific marker of COVID-19 infection [49].

The impression section, as mentioned above, includes the classification endorsed by the RSNA and other scientific societies into typical, indeterminate, atypical and negative [13], and the CO-RADS [10]. These items were set as “recommended” to address the radiologist to a well-defined conclusion, useful for the management of the clinical case. Both categorization systems have been included since these were reported in the literature at the same time and, to date, no further scientific validation has been performed on these systems to address a preferential use.

A final subsection of the impression section is dedicated to quantitative data on percentages of healthy parenchyma, emphysema, ground glass opacities, and consolidations. This last will not be of minor importance giving the rise on the market and in research of dedicated software for quantitative analysis. In line with the secondary aim of the structured report, the quantitative data are an additional component for a quantitative analysis in large clinical trials [50].

We believe that the combination of quantitative analysis data, lung volumes, and structured report items, with the help of deep learning techniques, will enable digital patient models to be extracted; therefore, the progression of the disease and the possible response to pharmacological treatments could be studied from these predictive and prognostic models. For this reason, many research groups have launched single and multicenter studies for the application of artificial intelligence on CT data [6, 33, 51–55].

At the time of the submission of this paper the radreport.org website did not include any COVID-19 structured report; however, a COVID-19 structured report is now available, and there are some differences with the one proposed by SIRM [56]. The SIRM report allows to classify the patient’s on the basis of a positive RT-PCR test or the clinical suspect of COVID-19, the severity of symptoms, and the pre-test probability of COVID-19, according to the key components of common clinical scenarios indicated by the statements by the Fleischner Society [5]. Meanwhile, the section of parenchymal findings has a similar content in both reports, but different structure; the SIRM report includes vascular findings, such as the caliber of the pulmonary artery, the pulmonary embolism, and potential vascular changes in the parenchyma. Moreover, in the impression section, the SIRM report includes also the CO-RADS classification and a quantification scheme for lung volume, which may have a value for statistical and research purposes.

## Conclusions

The proposed structured report could be of help both for expert radiologists, but also for the less experienced who are faced with the management of these patients. The compliance with the MRRT standard defined by RSNA allows it to share the structured report in HTML (HyperText Mark-up Language) for an easy implementation in radiological information systems. It can also be edited and modified for an adaptation to local clinical practice.

The structured report is conceived as a guideline, to suggest and recommend the key items/findings of chest CT in COVID-19 pneumonia.

## Abbreviations

ACR: American College of Radiology
BSTI: British Society of Thoracic Imaging
CO-RADS: Coronavirus reporting and data systems
COVID-19: Coronavirus 2019
COVID-RADS: Coronavirus reporting and data systems
CXR: Chest X-ray
ESR: European Society of Radiology
ESTI: European Society of Thoracic Imaging
HTML: Hypertext markup language
IHE: Integrating Healthcare Enterprise
MRRT: Management of Radiology Report Template
RSNA: Radiological Society of North America
SIRM: Italian Society of Medical and Interventional Radiology
SPSS: Statistical package for social science
WHO: World Health Organization
